# UPLC-QTOF-MS/MS and GC-MS Characterization of Phytochemicals in Vegetable Juice Fermented Using Lactic Acid Bacteria from Kimchi and Their Antioxidant Potential

**DOI:** 10.3390/antiox10111761

**Published:** 2021-11-04

**Authors:** Moeun Lee, Jung Hee Song, Eun Ji Choi, Ye-Rang Yun, Ki Won Lee, Ji Yoon Chang

**Affiliations:** 1 Research and Development Division, World Institute of Kimchi, Gwangju 61755, Korea; mon0007@wikim.re.kr (M.L.); jhsong@wikim.re.kr (J.H.S.); ejchoi@wikim.re.kr (E.J.C.); yunyerang@wikim.re.kr (Y.-R.Y.); 2 Biomodulation Major, Department of Agricultural Biotechnology, Seoul National University, Seoul 08826, Korea; 3 Advanced Institutes of Convergence Technology, Seoul National University, Suwon 16229, Korea

**Keywords:** antioxidant activities, functional foods, *Companilactobacillus allii* WiKim39, *Lactococcus lactis* WiKim0124, fermented vegetable juice

## Abstract

This study aims to investigate fermentative metabolites in probiotic vegetable juice from four crop varieties (*Brassica oleracea* var. *capitata*, *B. oleracea* var. *italica*, *Daucus carota* L., and *Beta vulgaris*) and their antioxidant properties. Vegetable juice was inoculated with two lactic acid bacteria (LAB) (*Companilactobacillus allii* WiKim39 and *Lactococcus lactis* WiKim0124) isolated from kimchi and their properties were evaluated using untargeted UPLC-QTOF-MS/MS and GC-MS. The samples were also evaluated for radical (DPPH^•^ and OH^•^) scavenging activities, lipid peroxidation, and ferric-reducing antioxidant power. The fermented vegetable juices exhibited high antioxidant activities and increased amounts of total phenolic compounds. Fifteen compounds and thirty-two volatiles were identified using UPLC-QTOF-MS/MS and GC-MS, respectively. LAB fermentation significantly increased the contents of d-leucic acid, indole-3-lactic acid, 3-phenyllactic acid, pyroglutamic acid, γ-aminobutyric acid, and gluconic acid. These six metabolites showed a positive correlation with antioxidant properties. Thus, vegetable juices fermented with WiKim39 and WiKim0124 can be considered as novel bioactive health-promoting sources.

## 1. Introduction

The use of food to improve health and reduce disease risk has gained importance in nutritional science and clinical studies and is important when considering the increasing cost of healthcare, steady increase in lifespan, and the desire to improve quality of life [1]. The functional food market is expanding, with a significant demand for prebiotics, probiotics, synbiotics, and products fortified with probiotics [2]. Although fermented dairy products are the most representative probiotics on the food market, interest in developing functional non-dairy probiotics as an alternative for consumers who wish to limit dairy consumption, such as people with lactose intolerance or vegan diets [3], has grown.

Kimchi, a traditional Korean fermented vegetable product, is a great source of probiotic LAB [4]; its health-related benefits are globally acknowledged [5]. The potential use of kimchi LAB as functional probiotics is continuously being investigated; kimchi LAB can be a good source from a pharmaceutical perspective by increasing its antioxidant and anticancer activities [6].

Vegetable juices are a tasty and healthy option for people of all ages. Vegetables are a good source of LAB strains [7]. We prepared vegetable juice (VJ) from cabbage (*Brassica oleracea* var. *capitata*), broccoli (*B. oleracea* var. *italica*), carrot (*Daucus carota* L.), and beetroot (*Beta vulgaris*), which are good sources of dietary fiber, sugars, phytochemicals, ascorbate, and phenolic compounds. These ingredients have been reported as biologically active compounds and encourage LAB growth [8,9,10,11]. Because vegetables contain beneficial nutrients, VJ may serve as an ideal food matrix for developing probiotic products. Furthermore, VJs fortified with LAB feature enhanced flavor and functionally bioactive substances [12]. Previous studies have confirmed the effects of probiotics on gastrointestinal and allergic diseases, immune modulation [13], the prevention of various types of cancer [14], and important roles in maintaining a healthy gut microbiome and prevention of several metabolic and cardiovascular diseases [15]. Fermented foods and beverages based on vegetable substrates are widely reported for their health-promoting properties, but are insufficiently studied, and their health claims are not supported by reliable scientific evidence [16].

A comprehensive evaluation of the fermentation activity promoted by LAB and the total metabolites in VJ are unknown. We fermented VJ using *C. allii* WiKim39 or *L. lactis* WiKim0124 isolated from kimchi. A metabolomics approach, using ultra-performance liquid chromatography quadrupole time-of-flight tandem mass spectrometry (UPLC-QTOF-MS/MS) and gas chromatography-mass spectrometry (GC-MS), was employed for the first time to identify, quantify, and compare the phytochemicals present in VJ fermented by LAB, and to analyze the correlation between these metabolites and their in vitro antioxidant properties.

## 2. Material and Methods

### 2.1. Bacterial Strains and Culture Conditions

The LAB strains *C. alli* WiKim39 (GenBank ID: NR_159087.1) and *L. lactis* WiKim0124 (GenBank ID: MZ424472.1) isolated from kimchi were evaluated in this study. The strains were stored at −80 °C in De Man, Rogosa, and Sharpe (MRS) agar (Difco; Detroit, MI, USA) containing 15% glycerol (*v*/*v*). For activation, 1% (*v*/*v*) of the culture was added to 10 mL of MRS broth and incubated at 30 °C for 24 h. The resulting suspension was used as an inoculum for 50 mL of MRS broth and incubated at 30 °C for 24 h. The cells were collected by centrifugation (6000× *g*, 10 min, 4 °C) for further use.

### 2.2. Preparation and Fermentation of VJ

The VJ was prepared using cabbage (*B. oleracea* var. *capitata*), broccoli (*B. oleracea* var. *italica*), carrot (*D. carota* L.), and beetroot (*B. vulgaris*), harvested from Jeju (Korea) in 2020. The vegetables were washed with tap water and completely dried. The VJ samples were prepared by mixing crushed vegetables and purified water (*w*/*v*) in the following ratio: 12% cabbage, 12% carrot, 12% broccoli, 10% beetroot, and 54% purified water. This formulation was determined by a preliminary sensory analysis based on taste (data not shown). The samples were extracted using pressurized hot water extraction at 107 °C for 2 h in a 5-ton stainless steel tank and then filtered with a 50-μm housing filter (Woosung Magnet Co., Ltd., Gimhae, Korea). The initial °Brix of the VJ samples was adjusted—5.5 °Brix for *C. alli* WiKim39 (VJ + WiKim39) and 10.0 °Brix for *L. lactis* WiKim0124 (VJ + WiKim0124) fermentation, respectively, with food-grade glucose according to optimal cell productivity (CFU/mL, data not shown). The VJ samples were sterilized at 98 °C for 10 min and filtered through a 100-μm filter (Woosung Magnet Co., Ltd.). After the VJ had cooled to room temperature, 10^7^ CFU/mL of each strain was used to inoculate the sterilized VJ and fermented at 30 °C for 48 h. For the in vitro experiments, the fermented samples were heat-killed at 95 °C for 10 min. This suspension was freeze-dried and stored at −70 °C until use. The non-inoculated VJ (pH 5.3 ± 0.2, 5.0 °Brix) was used as the control. All the samples were prepared in GMP (Good-Manufacturing-Practice)-compliant systems, which are the most suitable for food and drug applications.

### 2.3. Proximate Composition and Total Phenolic and Flavonoid Concentrations

The moisture, ash, crude protein, crude fat, and crude fiber content of the fermented and unfermented VJ samples were determined using the method recommended by the AOAC (2000). The carbohydrate content was calculated using the following formula: carbohydrates (%) = 100% − (moisture content (%) + crude fat content (%) + crude protein content (%) + ash content (%) + crude fiber (%)). The total phenolics (TP) were analyzed spectrophotometrically using the modified Folin–Ciocalteu method. The concentration of total phenolic compounds was expressed as gallic acid equivalent (µg GAE/mg). The total flavonoids (TF) were determined using the aluminum chloride colorimetric method with slight modifications and were expressed as catechin equivalent (µg CE/mg) [17].

### 2.4. Antioxidant Properties

#### 2.4.1. 2,2-Diphenyl-1-Picrylhydrazyl Radical Scavenging Activity

The activity of 2,2-Diphenyl-1-picrylhydrazyl radical (DPPH^•^) scavenging was determined as described in previous studies [18,19], with slight modifications. The samples (VJ, VJ + WiKim39, and VJ + WiKim0124) were dissolved in distilled water to a concentration of 100 mg/mL (initial concentration). Next, 10 μL of sample was added to the 180 μL of 0.2 mM DPPH solution and kept in the dark at 25 °C for 30 min. The absorbance was measured at 517 nm with ascorbic acid as the standard antioxidant (50 μg/mL, initial concentration). All the measurements were performed in triplicate. The DPPH^•^ scavenging activity was calculated using the equation below:

DPPH^•^ scavenging activity = (A_Blank_ − A_Sample_)/A_Blank_ × 100

where A_Sample_ is the absorbance in the presence of the sample and A_Blank_ is the absorbance of the distilled water control in the absence of the sample. DPPH radical scavenging activity was expressed as IC_50_ value and with ascorbic acid (vitamin C) comparison. All the measurements were performed in triplicate.

#### 2.4.2. Hydroxyl Radical Scavenging Activity

The assay was conducted based on the production of the hydroxyl radical (OH^•^) from a Fenton reaction between ferrous ions and hydrogen peroxide [20]. A total of 1 mL of sample (100 mg/mL, initial concentration) was mixed with 1 mL H_2_O_2_ (0.025%, *w*/*v*), 1 mL sodium salicylate (9 mM), and 1 mL FeSO_4_ (9 mM). The reaction mixture was incubated at 37 °C for 1 h and cooled. The absorbance was measured at 536 nm with ascorbic acid as the standard antioxidant (50 μg/mL, initial concentration) and distilled water was used as a negative control. The OH^•^ scavenging activity was calculated using the following equation:

% OH^•^ scavenging rate = (A_Sample_ − A_Control_)/(A_Blank_ − A_Control_) × 100

where A_Sample_ is the absorbance in the presence of the sample, A_Control_ is the absorbance of the control in the absence of the sample, and A_Blank_ is the absorbance without the sample and the Fenton reaction system. All the measurements were performed in triplicate.

#### 2.4.3. Ferric Reducing Antioxidant Power

The ferric reducing antioxidant power (FRAP) assay was performed using a commercial kit (ab234626; Abcam, Hong Kong, China). After ferric-tripyridyltriazine (Fe^+3^-TPTZ) in the reaction mixture was converted to Fe^+2^ in an acidic environment (pH 3.6), the absorbance was measured at 594 nm. The quantification was performed using a calibration curve of ferrous sulfate and the results were expressed as Fe^2+^ equivalents (μM).

#### 2.4.4. Inhibition Rate of Lipid Peroxidation

The inhibition rate of lipid peroxidation in the samples was evaluated as the ability of biological fluids to inhibit the production of thiobarbituric acid reactive substances (TBARS) using the thiobarbituric acid (TBA) method, as described [21]. Butylated hydroxytoluene (BHT) (3 mM, initial concentration) was used as the reference. The inhibition rate of lipid peroxidation was calculated using the formula:

Inhibition rate of lipid peroxidation (%) = (A_Blank_ − A_Sample_/A_Blank_) × 100

where A_Sample_ is the absorbance in the presence of the sample and A_Blank_ is the absorbance of the control in the absence of the sample. All the measurements were performed in triplicate.

### 2.5. VJ Fermentation Metabolite Analysis

#### 2.5.1. UPLC-QTOF-MS/MS Profile

The sample replicates from the VJ fermentation were purified using the Sep-Pak C18 cartridge (Waters, Dublin, Ireland), as described in [22]. Next, the solutions were filtered through a 0.22-μm syringe filter. The purified samples were screened using UPLC-QTOF-MS/MS. The UPLC-QTOF-MS/MS system consisted of the Vanquish UPLC system (Thermo Fisher Scientific, San Jose, CA, USA) with a Waters CORTECS T3, C18 column (2.1 × 150 mm, 1.6 μm; Waters, Milford, MA, USA) connected to the TSQ Altis triple quadrupole mass spectrometer (Thermo Scientific, San Jose, CA, USA). Via an electrospray ionization (ESI) interface, the QTOF-MS/MS was used to complete the high-resolution experiment in the negative ion mode. The analytical detector was a Waters Acquity PDA detector, which was set to a wavelength range of 200–400 nm. The elution program for the UPLC separation used 10 mM ammonium acetate in water as eluent A and methanol as eluent B. The gradient elution program was as follows: 0–0.4 min; 15% B, 0.5–7 min; 15% B, 7–7.5 min; 15–100% B, 7.5–8 min; 100–15% B, and equilibration with 15% B for 5 min at a flow rate of 0.2 mL/min. The column was set at 45 °C, and the auto-sampler was maintained at 4 °C. The injection volume of each sample solution was 3 μL. The detailed experimental conditions are listed in Supplementary Table S1. The data acquisition and analysis were performed using Thermo Xcalibur™ software (Thermo Fisher Scientific, San Jose, CA, USA). The standard solutions of d-leucic acid (d-LA), indole-3-lactic acid (ILA), and 3-phenyllactic acid (3-PLA) were obtained from Sigma Aldrich (St. Louis, MO, USA).

#### 2.5.2. GC-MS Profile

For the derivatization, 100 μL of 20,000 ppm pyridine were added to 5 mg of each lyophilized sample and incubated at 30 °C for 90 min. Next, 100 μL of *N*,*O*-bis(trimethylsilyl)trifluoroacetamide with 1% trimethylchlorosilane solution and 20 μL of internal standard (1000 ppm fluoranthene) were added to the sample vial and incubated at 60 °C for 30 min. The samples were analyzed through GC-MS. GC chromatographic separations were achieved on a Thermo Scientific TRACE™ 1310 Gas Chromatograph with a single quadrupole mass spectrometer. The GC was equipped with a capillary column (Agilent, Palo Alto, CA, USA; DB-5MS 60 m × 0.25 mm × 0.25 μm) and run in full-scan mode (scan range 40–700 m/z with detector voltage 2160 V). The gas carrier was helium, with a flow rate of 1.5 mL/min. The transfer line was settled at 310 °C and 1 μL of the sample was injected. The oven temperature was fixed at 50 °C for 2 min, increased to 180 °C (rate 5 °C/min), held for 5 min, increased to 325 °C (rate 5 °C/min), and held for 10 min. The ion source was settled at 270 °C and the solvent delay was 4.5 min. Mass spectra were recorded in electronic impact mode at 70 eV, scanning within the 35–650 m/z range for the selection of appropriate electronic impact mass fragments for each analyte, with a scan rate of 6 scans/s. The standard solutions (pyroglutamic acid, γ-aminobutyric acid, and gluconic acid) were obtained from Sigma Aldrich (St. Louis, MO, USA).

### 2.6. Statistical Analysis

The statistical analysis was performed using Tukey’s honest significant difference (HSD) test carried out in the “agricolae” package of R (v3.3.2; https://www.r-project.org/, accessed on 31 December 2016) for group comparisons. Non-parametric correlation estimations (Spearman’s correlations) were performed using the corrplot function from the “PerformanceAnalytics” package in R. Results with *p* < 0.05 were considered statistically significant.

## 3. Results and Discussion

### 3.1. Proximate Composition and TP and TF Content

The moisture, ash, crude protein, crude fat, crude fiber, carbohydrate, TP, and TF content of freeze-dried fermented VJ are shown in Table 1. The fermented VJ contained high levels of carbohydrate (86.23–88.17%); moderate levels of moisture (6.66–7.05%); and low levels of crude protein (2.24–3.60%), ash (1.60–1.68%), crude fat (0.49–0.69%), and crude fiber (0.45–0.85%). Due to fermentation, the crude protein content was significantly higher in VJ + WiKim39 (3.20%) and VJ + WiKim0124 (3.06%). The increase in the protein content may have been due to the proteolytic enzymes produced during microbial fermentation or the result of synthesizing proteins by fermenting substrates that may have resulted in amino acid production [23]. The crude fat content significantly increased in the LAB-fermented VJs; VJ + WiKim0124 demonstrated the highest content, followed VJ + WiKim39, at 0.85% and 0.79%, respectively. The VJ demonstrated a carbohydrate content of 88.17%, followed by VJ + WiKim0124 (87.07%) and VJ + WiKim39 (86.23%). This may have been due to the possible bioconversion of carbohydrates to crude fat [24]. The crude fiber content significantly increased in the LAB-fermented groups; however, in the present study, the VJ samples were filtered using a 50 µm filter and the fiber content may have been affected in the filtrate. Therefore, it cannot be addressed in this study.

The TP and TF content in the samples are crucial because of their positive effect on the physiological processes, including antioxidant activities, anti-inflammatory properties, and cancer-risk reduction activity [25]. The TP ranged from 206.29 to 255.69 μg GAE/g. VJ + WiKim39 contained the highest level of TP, at 255.69 μg GAE/g, whereas VJ presented the lowest content, at 206.29 μg GAE/g. The TF ranged from 11.62 to 16.75 μg CE/g. The highest level of TF was found in VJ + WiKim39, at 16.75 μg CE/g, whereas VJ + WiKim0124 presented the lowest TF content, at 11.62 μg CE/g. The significant increase in TP in the LAB-fermented samples may have been due to the hydrolytic enzymes in the LAB strains that hydrolyze complex phytochemicals into simpler forms. The differences in phytochemical concentrations between LAB strains (WiKim39 and WiKim0124) may have been due to their individual adaptability and their ability to produce more hydrolytic enzymes [26]. An increase in TF was observed in VJ + WiKim39, which may have been due to the enzymatic breakdown of the complex polyphenols into simpler flavanol compounds during fermentation [27]. A significant decrease in TF was observed in VJ + WiKim0124, probably because of the depolymerization of high molecular weight phenolic compounds by polyphenol oxidase in the LAB strain [7].

### 3.2. Antioxidant Capacity of Fermented VJ

**Figure 1:**
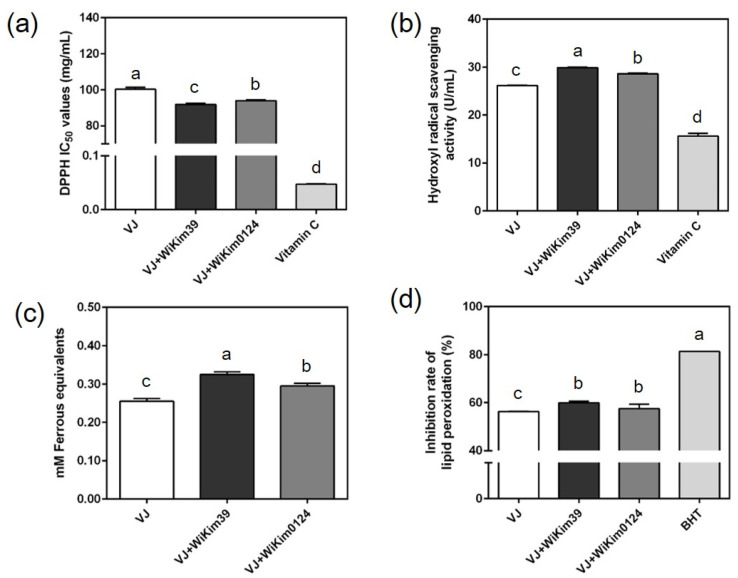
Antioxidant capacities of vegetable juice fermented with lactic acid bacteria. (**a**) DPPH radical scavenging activity, (**b**) hydroxyl radical scavenging activity, (**c**) ferric-reducing antioxidant power assay, (**d**) inhibition rate of lipid peroxidation assay (TBARS). At least triplicate analyses were performed. Different letters above the columns denote significant differences (*p* < 0.05). DPPH: 2,2-Diphenyl-1-picrylhydrazyl radical, TBARS: thiobarbituric acid reactive substances.

Metal ions (Fe^3+^) and free radicals are important oxidants involved in the pathogenic process of many chronic diseases, and good antioxidants should not only reduce oxidants, but also scavenge free radicals. Lipid oxidation is problematic in food systems. Lipid oxidation proceeds through a free-radical chain mechanism and is an indicator of oxidative damage in physiological systems, as many end products produce toxic compounds and are a major cause of diseases, such as atherosclerosis [28]. To evaluate the antioxidant capacity of fermented and non-fermented VJs, DPPH^•^ and OH^•^ scavenging, FRAP, and TBARS assays were used. We found distinct effects of fermentation on antioxidant activities. Compared to the non-fermented samples, the LAB-fermented samples generally displayed higher antioxidant capacities. As shown in Figure 1, VJ was more susceptible to lipid peroxidation than VJ + WiKim39 and VJ + WiKim0124 and featured lower DPPH^•^ and OH^•^ scavenging capacity. The quantification of antioxidant capacity with FRAP showed an increase in antioxidant capacity in VJ + WiKim39 and VJ + WiKim0124. The increased antioxidant activity may have been due to the ability to release active compounds from the VJ matrix during fermentation, which is thought to be associated with the production of phenolic compounds [29]. However, phytochemicals other than TP can also contribute to antioxidant activities. Therefore, we performed high-resolution LC-MS/MS and GC-MS to explore identify phytochemicals in fermented VJs.

### 3.3. Phytochemical Compounds Formed during VJ Fermentation

#### 3.3.1. UPLC-QTOF-MS/MS Characterization of Fermented VJ

**Figure 2:**
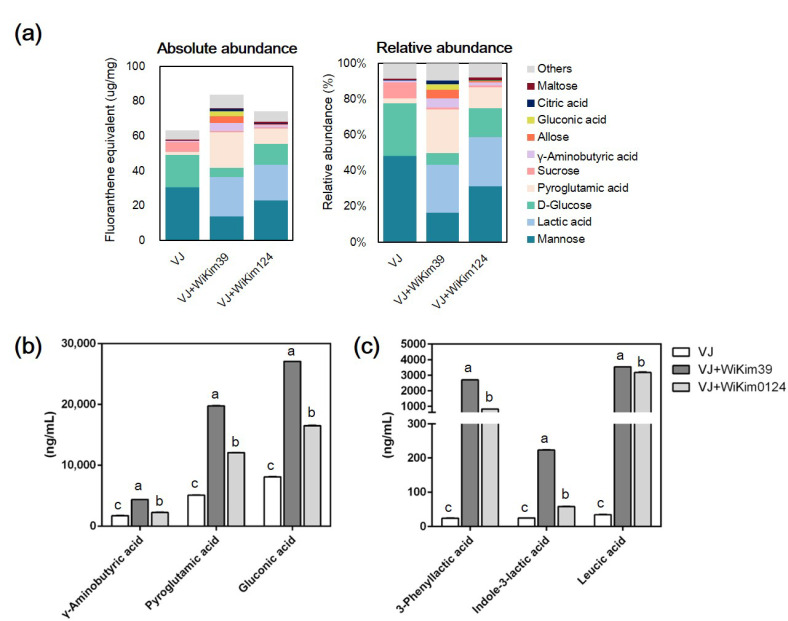
Identification of phytochemical profile in fermented or non-fermented vegetable juice. (**a**) GC-MS profile of the top 10, most abundant phytochemicals. (**b**,**c**) Quantification of significantly differentiated compounds from GC-MS or UPLC-QTOF-MS/MS using UPLC-QTOF-MS/MS. (Details of ion chromatograms are shown in Supplementary Figures S1 and S2). GC-MS: gas chromatography-mass spectrometry, UPLC-QTOF-MS/MS: ultra-performance liquid chromatography with quadrupole time-of-flight tandem mass spectrometry. Different letters denote significant differences (*p* < 0.05, ANOVA, Tukey-HSD).

A total of 12, 12, and 14 compounds were tentatively identified from VJ, VJ + WiKim39, and VJ + WiKim0124, respectively, by their exact masses, MS/MS spectra, and molecular formulas. As shown in Table 2, the metabolites consisted of organic acids, phenols, sugars, and miscellaneous compounds. Among these, the components detected only in the LAB-fermented products were d-LA, ILA, and 3-PLA; these compounds were further confirmed quantitatively by comparison with standard solutions (Figure 2C and Supplementary Table S3). All the fermented VJs exhibited significantly higher concentrations than their respective non-fermented VJ. VJ + WiKim39 contained the highest levels of d-LA, ILA, and 3-PLA, at 3535.63, 222.68, and 2687.33 ng/mL, whereas VJ contained the lowest at 34.35, 24.18, and 23.18 ng/mL, respectively. The first identification step allowed the subsequent quantification of LAB-derived influencing factors and aimed to elucidate the expected impact of WiKim39 and WiKim0124 on the production of bioactive substances through VJ fermentation.

The leucine derivative metabolite, d-LA (2-hydroxyisocaproic acid), is produced during *Lactobacillus*-mediated fermentation and is also found in human tissues. Reportedly, d-LA exhibited anti-inflammatory effects in a murine *Candida* biofilm model [30]. Because d-LA can improve muscle recovery by increasing protein synthesis, it can be used as a food additive to promote muscle growth. In addition, d-LA is used as an antibacterial agent and as a treatment for infected body cavities and sinuses. [31]. ILA is a microbial tryptophan-derived indole compound, which acts as an antioxidant and free-radical scavenger [32]. ILA also promotes the inflammatory control and neuronal developmental processes [33]. The phenolic acid phytochemical, 3-PLA, is synthesized during phenylalanine metabolism in LAB. High levels of 3-PLA are produced during LAB-mediated fermentation; 3-PLA has broad antibacterial activity against bacteria and fungi, making it a promising naturally occurring substance that can be used for extending the shelf life of food. Furthermore, 3-PLA is non-toxic to cells and animals, and the dietary supplementation of 3-PLA in animal models enhances its immunomodulatory effects [34]. Our LAB-fermented VJs were rich in these compounds, providing clues to their functional properties and potential relevance.

#### 3.3.2. GC-MS Characterization of Fermented VJ

A total of 29, 31, and 31 volatile constituents were tentatively identified from VJ, VJ + WiKim39, and VJ + WiKim0124, respectively, through GC-MS. The compounds were categorized as amino acids, fatty acids, organic acids, sugars, and miscellaneous (Supplementary Table S2). To determine differences in the composition of volatiles between LAB-fermented and non-fermented VJ products, the 10 most abundant volatiles in the samples (Figure 2A), sugars (12.42–26.27%), organic acids (0.61–13.33%), and amino acids (1.08–12.27%) were dominant in the total proportion. Sugars (mannose, d-glucose, and sucrose) showed the highest abundance ratio in the VJ; on the other hand, organic acids and amino acids were increased in fermented VJ. The reduction in sugars during fermentation was a result of bioconversion to organic acids and the use of LAB strains for propagation [35]. In particular, VJ + WiKim39 significantly reduced the absolute amount of sugar while increasing the content of organic acids and amino acids. In these data, pyroglutamic acid (PGA), γ-aminobutyric acid (GABA), and gluconic acid (GA) were significantly increased during LAB fermentation. These compounds were quantitatively confirmed using standard solutions (Figure 2B and Supplementary Table S3). VJ + WiKim39 contained the highest levels of GABA, PGA, and GA, while VJ contained the lowest. An obvious difference in trend was observed between VJ and fermented VJ.

PGA (oxoproline) and GABA are widely recognized as important biomarkers for diseases and effects of drugs [36]. PGA is a glutamic acid derivative lacking a water molecule; it has been shown to have pharmacological properties for the regulation of amino acid transport and to accelerate the removal of potentially harmful amino acids [37]. Thus, PGA may serve as a therapeutic target in glutamate cytotoxicity, which is toxic to the brain at very low concentrations and leads to ischemia or traumatic brain injury [38]. GABA is present in many vegetables and fruit. This amino acid has therapeutic effects on autoimmune diseases, including neurodegenerative diseases [39]. Moreover, GABA reduces blood pressure [40], offers neuroprotective effects, modulates inflammation [41] and produces antioxidant activity [42]. GA and its salts are produced by the oxidation of the first carbon of β-d-glucose to a carbonyl group by chemical or enzymatic transformation. GA and its salts feature a high ability to form water-soluble complexes with divalent or trivalent metal ions, making them suitable for various pharmaceutical, food, and other industrial applications [43]. GA and its derivatives could potentially be effective as intestinal regulators or inhibitors of intestinal decay in both animals and humans. Research in this area has focused on the antioxidant properties of GA derivatives [44].

#### 3.3.3. Comparative Analysis of Fermented VJ Phytochemicals and Their Antioxidant Properties

**Figure 3:**
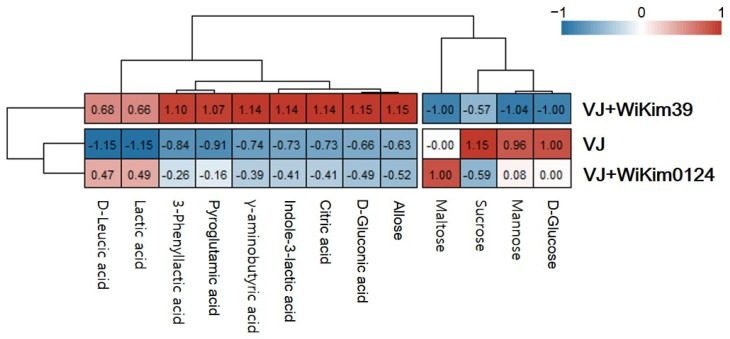
Heatmap. Distribution and concentration of phytochemicals among samples. Red boxes mean higher concentrations. Blue boxes mean lower concentrations.

**Figure 4:**
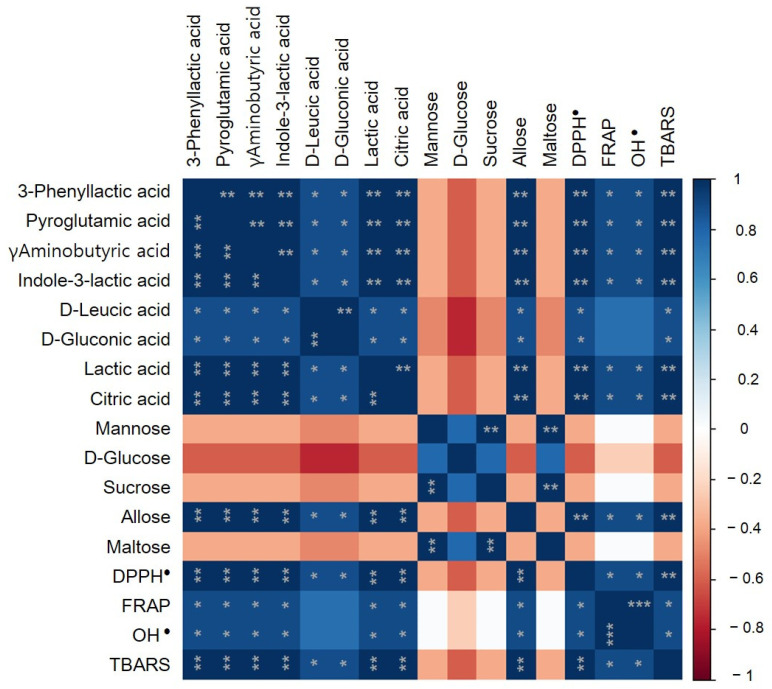
Spearman’s rank correlation. Relationships between antioxidant assays and metabolites. Spearman’s rank correlation coefficient ranged from 1.0 to −1.0 and corresponded to a strongly positive or negative correlation, respectively (* *p* < 0.05; ** *p* < 0.01; *** *p* < 0.005).

To intuitively represent the difference between non-fermented and fermented VJs, a heat map (Figure 3) was produced, along with hierarchical clusters based on the mean values of component concentrations from LC-QTOF-MS/MS and GC-MS. For the columns and rows, the clustering method was used based on the mean. The heat map shows the changes in the relative chemical composition of each type, showing differences that could be related to different LAB strains. Two clusters were observed in rows and columns, emphasized by hierarchical clustering. According to this analysis, VJ and VJ + WiKim0124 displayed similar features, with a relatively high amount of sugars. The overall sugar content was high in VJ, and only the mannose content was high in VJ + WiKim0124. By contrast, in VJ + WiKim39, the content of phytochemicals, other than sugars, was high. This cluster demonstrated a significant difference in crucial metabolites between VJ + WiKim39 and other samples. The clusters shown in the row represent the distribution patterns among the metabolites. In the fermented VJ samples, the sugar content decreased and the amount of organic acids and amino acids increased. Figure 4 shows Spearman’s rank correlations with crucial metabolites and antioxidant properties. The composite metabolites, excluding sugar, demonstrated a significant positive correlation with antioxidant capacities. In particular, a strong positive correlation with DPPH and TBARS (*p* < 0.01) was seen. The d-LA and GA displayed reduced positive correlation with antioxidant capacities relative to the others. Studies have found a positive correlation between antioxidant capacity and polyphenol compounds in plant-based foods, such as fruits and vegetables [45]. However, in this study, the variety and content of individual phytochemicals contributed significantly to differentiating the fermented VJ beverages.

In addition to the phenolic acids in the fermented VJs, organic acids, and amino acids may correlate with antioxidant activity.

## 4. Conclusions

To verify the health-promoting effects of LAB-fermented VJ, metabolites generated through LAB fermentation were analyzed to establish the basis for a functional drink. We profiled VJ, VJ + WiKim39, and VJ + WiKim0124 using LC-QTOF-MS/MS and GC-MS and investigated the correlation between antioxidant capacity and the phytochemicals in fermented and non-fermented VJs. Significant metabolite changes were found in the LAB-fermented VJs and a significant positive correlation between compounds and their antioxidant capacities was observed. Based on these results, six functional active ingredients were identified, including three organic acids, one phenol, and two amino acids. More active substances were detected in VJ + WiKim39 than in VJ + WiKim0124. The results demonstrated a positive effect of LAB fermentation on antioxidant activities. Our results are promising and suggest that lactic acid bacteria *C. allii* WiKim39 and *L. lactis* WiKim0124 isolated from kimchi are promising for the development of probiotic beverages; thus, they could be used as functional starter cultures to increase the antioxidant activity of plant-based foods during fermentation. Moreover, further studies on phenolic compounds and flavonoid content variation during the VJ fermentation process will be necessary in the future.

## Figures and Tables

**Table 1:** Proximate composition, TP and TF content of non-fermented or fermented vegetable juice with LAB.

Parameters (g/100 g)	VJ	VJ + WiKim39	VJ + WiKim0124
Moisture	7.05 ± 0.25 ^a^	7.01 ± 0.15 ^a^	6.66 ± 0.21 ^b^
Ash	1.60 ± 0.12	1.68 ± 0.11	1.67 ± 0.15
Crude protein	2.24 ± 0.01 ^c^	3.60 ± 0.05 ^a^	3.06 ± 0.02 ^b^
Crude fat	0.49 ± 0.15 ^a^	0.69 ± 0.12 ^b^	0.69 ± 0.01 ^a^
Carbohydrate *	88.173 ± 0.16	86.23 ± 0.14	87.07 ± 0.05
Crude fiber	0.45 ± 0.15 ^b^	0.79 ± 0.01 ^b^	0.85 ± 0.01 ^a^
TP (µg Garlic acid equivalents)	206.29 ± 1.44 ^c^	255.69 ± 2.44 ^a^	240.52 ± 0.72 ^b^
TF (µg Catechin equivalents)	12.67 ± 0.09 ^b^	16.75 ± 0.13 ^a^	11.62 ± 0.15 ^c^

Values are given as mean ± standard deviation (n = 3) in dry matter basis. Different superscripts in the same row are significantly different (*p* < 0.05, ANOVA, Tukey-HSD). * Carbohydrate (%) = 100 − (% ash + % crude protein + % crude fat + % crude fiber).

**Table 2:** Tentatively identified compounds from probiotic vegetable juice samples by UPLC-QTOF-MS/MS under negative ion mode.

Class	Tentative Identification	RT (min)	Molecular Formula	Molecular Weight	Molecular Ion [M − H]^−^ (*m*/*z*)	Error (ppm)	VJ	VJ + WiKim39	VJ + WiKim0124
Organic acids	Citric acid	1.52	C_6_H_8_O_7_	192	191.02	0.263	● *	●	●
	3-*O*-Coumaroylquinic acid	14.09	C_16_H_18_O_8_	338.1	337.094	2.856	●	●	●
	D-Leucic acid ^a^	12.31	C_6_H_12_O_3_	132.1	131.072	5.655	ND	●	●
	1,8-nonanedioic acid	23.55	C_9_H_16_O_4_	188.1	187.098	3.655	●	●	●
	Indole-3-lactic acid ^a^	17.84	C_11_H_11_NO_3_	205.1	204.067	1.636	ND	●	●
	Malic acid	1.25	C_4_H_6_O_5_	134	133.015	2.128	●	ND	ND
Phenols	Caffeic acid	10.92	C_9_H_8_O_4_	180	179.036	1.978	●	ND	●
	5-Hydroxyquinoline	18.28	C_9_H_7_NO	145.1	144.046	4.951	●	●	●
	3-Phenyllactic acid ^a^	15.12	C_9_H_10_O_3_	166.1	165.056	2.802	ND	●	●
	Salicylic acid	7.39	C_7_H_6_O_3_	138	137.024	0.054	●	●	●
	Neochlorogenic acid	7.77	C_16_H_18_O_9_	354.1	353.089	2.492	●	●	●
	Ferulic acid	17.69	C_10_H_10_O_4_	194.1	193.051	1.646	●	ND	●
Sugar	Sucrose	1.14	C_12_H_22_O_11_	342.1	341.109	2.319	●	●	●
	D-Tagatose	1.09	C_6_H_12_O_6_	180.1	179.057	2.029	●	●	●
Miscellaneous	9-(2,3-dihydroxypropoxy)-9-oxononanoic acid	23.29	C_12_H_22_O_6_	262.1	261.135	1.907	●	●	●

* Filled circles indicate presence of metabolites. ^a^ The compound was further identified using the corresponding standard compound ND, not detected.

## Data Availability

The data presented in this study are available in this article.

## Supplementary Materials

The following are available online at https://www.mdpi.com/article/10.3390/antiox10111761/s1. Figure S1: total Ion Chromatogram obtained using UPLC-QTOF-MS/MS. Quantification of significantly differentiated compounds from UPLC-QTOF-MS/MS profile. UPLC-QTOF-MS/MS: ultra-performance liquid chromatography with quadrupole time-of-flight tandem mass spectrometry. Figure S2: total Ion Chromatogram obtained by UPLC-QTOF-MS/MS. Quantification of significantly differentiated compounds from GC-MS profile. GC-MS: gas chromatography-mass spectrometry, UPLC-QTOF-MS/MS: ultra-performance liquid chromatography with quadrupole time-of-flight tandem mass spectrometry. Supplementary Table S1: detailed analytical conditions of UPLC-QTOF-MS/MS. Supplementary Table S2: tentatively identified compounds from probiotic vegetable juice samples by GC-MS, Supplementary Table S3: quantitative characterization of significant phytochemical compounds in VJ samples by UPLC-QTOF-MS/MS.
