# Supplementary material for: UPLC-QTOF-MS/MS and GC-MS Characterization of Phytochemicals in Vegetable Juice Fermented Using Lactic Acid Bacteria from Kimchi and Their Antioxidant Potential
Source: Antioxidants (Basel). 2021 Nov 4;10(11):1761. doi: 10.3390/antiox10111761 (PMC8614894; doi:10.3390/antiox10111761)
Supplement: Supplementary file 1 [file antioxidants-10-01761-s001.zip › antioxidants-1442870-supplementary.pdf]

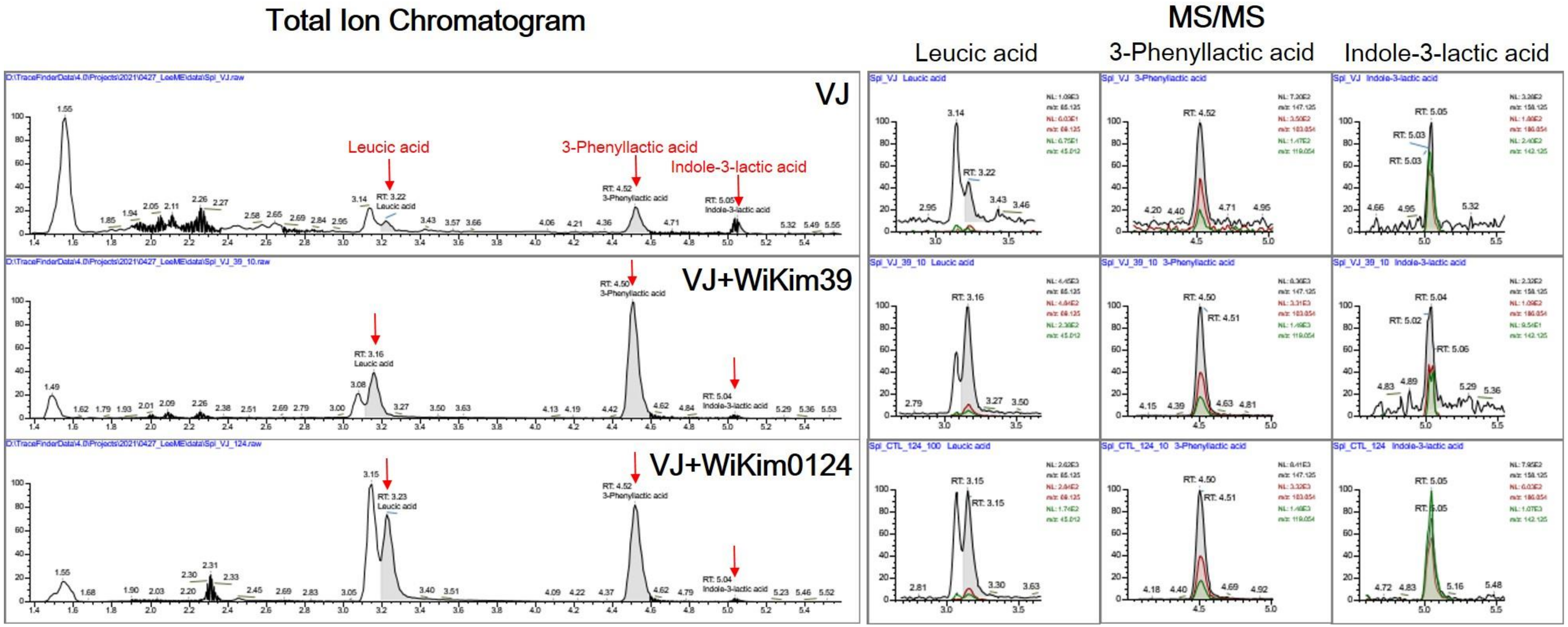

Supplementary Fig. S1. Total Ion Chromatogram obtained using UPLC-QTOF-MS/MS. Quantification of significantly differentiated compounds from UPLC-QTOF-MS/MS profile.

UPLC-QTOF-MS/MS: ultra-performance liquid chromatography with quadrupole time-of-flight tandem mass spectrometry

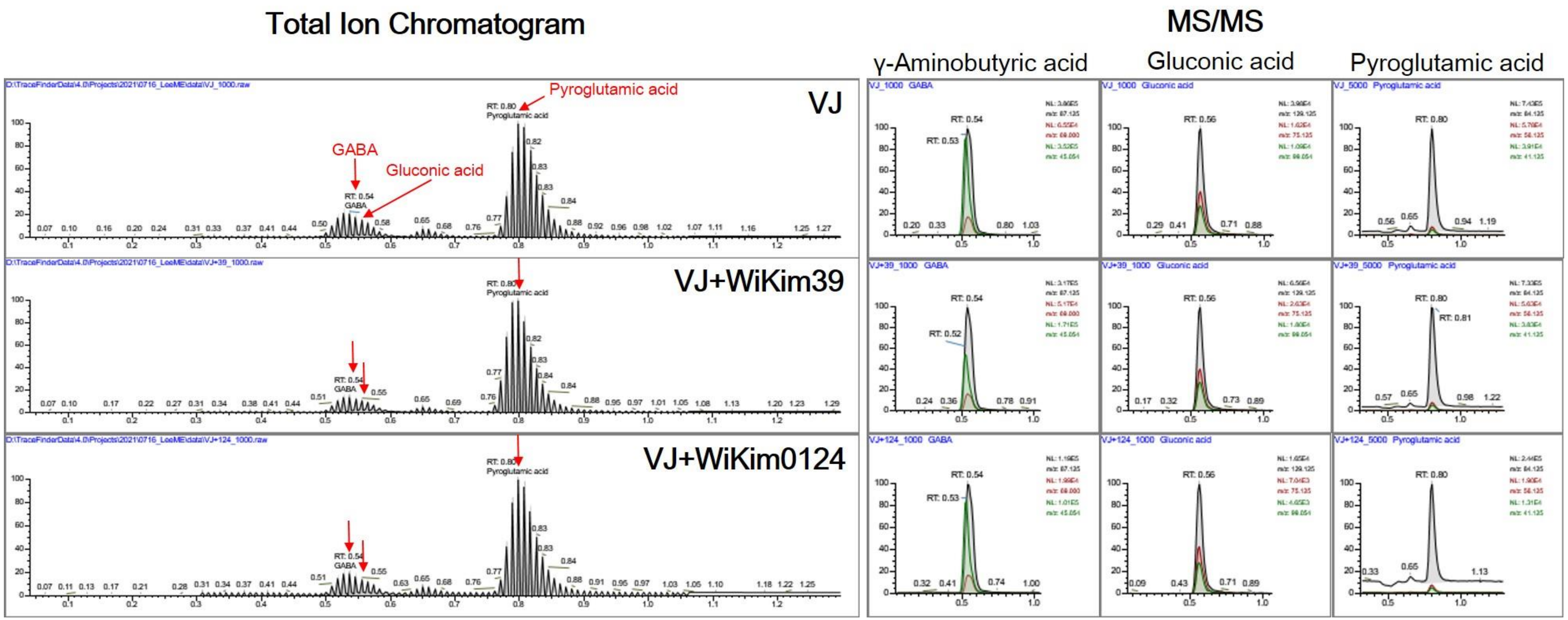

Supplementary Fig. S2. Total Ion Chromatogram obtained by UPLC-QTOF-MS/MS. Quantification of significantly differentiated compounds from GC-MS profile.

GC-MS: gas chromatography-mass spectrometry, UPLC-QTOF-MS/MS: ultra-performance liquid chromatography with quadrupole time-of-flight tandem mass spectrometry

Supplementary Table S1. Detailed analytical conditions of UPLC-QTOF-MS/MS.

Instrument	TSQ Altis triple-quadrupole mass spectrometer (Thermo Scientific, USA)
Ionization	Electrospray ionisation (H-ESI)
Negative Ion / Positive Ion (V)	2500 / 3500
Sheath Gas (Arb)	50
Aux Gas (Arb)	10
Sweep Gas (Arb)	1
Ion Transfer Tube Temp (°C)	325
Vaporizer Temp (°C)	350
Scan type	Selected reaction monitoring (SRM)
Polarity	Negative / Positive

Supplementary Table S2. Tentatively identified compounds from probiotic vegetable juice samples by GC-MS.

Class	Tentative identification	RT (min)	Molecular Formula	Molecular Weight	Fluoranthene equivalent ug/mg		
					VJ	VJ+ WiKim39	VJ+ WiKim0124
Amino acids	Alanine_2TMS	17.37	$C_3H_7NO_2$	116	0.013	0.134	0.196
	Valine_2TMS	23.17	$C_5H_{11}NO_2$	142	0.021	0.083	0.121
	Glycine_3TMS	23.38	$C_2H_5NO_2$	174	0.096	0.655	0.175
	Pyroglutamic acid_2TMS[a]	29.09	$C_5H_7NO_3$	156	1.558	20.438	8.650
	γ-Aminobutyric acid_3TMS[a]	29.30	$C_4H_9NO_2$	174	0.592	4.362	1.300
	Proline_2TMS	39.74	$C_5H_9NO_2$	217	0.004	0.007	0.246
	Serine_2TMS	43.38	$C_3H_7NO_3$	103	ND	0.341	0.092
Fatty acids	Propanoic acid_1TMS	16.52	$C_3H_6O_2$	147	0.017	0.469	0.004
	Stearic acid_1TMS	45.44	$C_{18}H_{36}O_2$	319	0.338	0.421	0.063
	Palmitic acid_1TMS	51.79	$C_{16}H_{32}O_2$	217	0.692	0.445	0.071
	Oleic acid_1TMS	55.63	$C_{18}H_{34}O_2$	117	0.063	0.072	0.013
Organic acids	Lactic acid_2TMS	16.00	$C_3H_6O_3$	147	0.071	22.645	20.433
	Malic acid_3TMS	22.23	$C_4H_6O_5$	174	0.963	ND	ND
	Glyceric acid_3TMS	23.94	$C_3H_6O_4$	147	0.033	0.062	0.071
	Fumaric acid_2TMS	24.57	$C_4H_4O_4$	245	0.021	0.121	0.033

	Citric acid_4TMS	28.09	$C_6H_8O_7$	147	0.058	1.679	0.342
	Gluconic acid_6TMS	48.50	$C_6H_{12}O_7$	147	0.071	2.562	0.304
	Glycolic acid_2TMS	42.96	$C_2H_4O_3$	103	0.029	0.403	0.092
	Succinic acid_2TMS	50.60	$C_4H_6O_4$	117	0.054	0.821	0.225
Sugar	Fructose_5TMS	22.01	$C_6H_{12}O_6$	116	0.029	0.410	0.100
	Arabinose_4TMS	33.32	$C_5H_{10}O_5$	103	0.013	0.114	0.042
	Inositol_6TMS	37.80	$C_6H_{12}O_6$	292	0.017	0.155	0.029
	Maltose_8TMS	40.56	$C_{12}H_{22}O_{11}$	147	0.529	0.138	0.921
	Mannose_5TMS	44.90	$C_6H_{12}O_6$	205	30.417	13.617	23.067
	Myoinositol_6TMS	46.80	$C_6H_{12}O_6$	217	0.225	0.800	0.413
	D-Glucose_5TMS	47.81	$C_6H_{12}O_6$	204	18.671	5.559	12.104
	Fructofuranose_5TMS	49.58	$C_6H_{12}O_6$	217	ND	0.162	0.017
	Allose_6TMS	53.25	$C_6H_{12}O_6$	319	0.088	4.045	0.342
	Mannitol_6TMS	56.29	$C_6H_{14}O_6$	117	ND	0.579	0.017
	Sucrose_8TMS	63.71	$C_{12}H_{22}O_{11}$	361	5.746	0.776	0.7179
Miscellaneous	Ribonic acid_5TMS	20.76	$C_5H_{10}O_6$	144	0.013	0.183	0.025
	Aminoethanol_3TMS	23.58	C_2H_7NO	147	0.133	0.645	0.229

[a], The compound was further identified using the corresponding standard compound. ND, not detected.

Supplementary Table S3. Quantitative characterization of significant phytochemical compounds in VJ samples by UPLC-QTOF-MS/MS.

Compound name	RT (min)	Ionization (ESI^-/ESI^+)	Molecular ion (m/z)	MSMS products ions (m/z)	Collision energy (V)	(ng/mL)		
						VJ	VJ+ WiKim39	VJ+ WiKim0124
D-Leucic acid	3.01	[M-H]-	131	45.01<br>69.13<br>85.13	13.76<br>20.42<br>12.25	34.18 ± 0.25	3532.82 ± 3.98	3147.06 ± 4.16
Indole-3-lactic acid	3.95	[M-H]-	204	142.13<br>158.13<br>186.05	18.48<br>15.15<br>13.47	24.59 ± 0.58	226.34 ± 5.18	59.19 ± 1.15
3-Phenyllactic acid	4.26	[M-H]-	165	103.05<br>119.05<br>147.13	15.70<br>16.79<br>11.23	23.59 ± 0.58	2693.67 ± 8.96	819.57 ± 0.62
γ-Aminobutyric acid	0.54	[M+H]+	104.09	45.05<br>69.00<br>87.13	21.01<br>15.49<br>10.52	1728.70 ±13.90	4334.11 ±16.33	2245.83 ±56.45

Gluconic acid	0.56	[M-H]-	195	75.13<br>99.05<br>129.13	17.76<br>14.14<br>12.20	8080.95 ±34.90	27073.61 ±18.27	16500.66 ±57.78
Pyroglutamic acid	0.8	[M+H]+	130.04	41.13<br>56.13<br>84.13	21.81<br>24.12<br>13.00	5071.72 ±12.39	19741.34 ±65.51	12061.11 ±11.83
